# Phosphatidylcholine-specific phospholipase C inhibition reduces HER2-overexpression, cell proliferation and *in vivo* tumor growth in a highly tumorigenic ovarian cancer model

**DOI:** 10.18632/oncotarget.18992

**Published:** 2017-07-05

**Authors:** Luisa Paris, Franca Podo, Francesca Spadaro, Laura Abalsamo, Maria Elena Pisanu, Alessandro Ricci, Serena Cecchetti, Luisa Altabella, Maria Buoncervello, Ludmila Lozneanu, Marina Bagnoli, Carlo Ramoni, Silvana Canevari, Delia Mezzanzanica, Egidio Iorio, Rossella Canese

**Affiliations:** ^1^ Department of Cell Biology and Neurosciences, Istituto Superiore di Sanità, 00161, Roma, Italy; ^2^ Department of Hematology, Oncology and Molecular Medicine, Istituto Superiore di Sanità, 00161, Roma, Italy; ^3^ Department of Experimental Oncology and Molecular Medicine, Fondazione IRCCS Istituto Nazionale dei Tumori, 20133, Milano, Italy; ^4^ Department of Histology, University of Medicine and Pharmacy “Grigore T. Popa”, 700115, Iasi, Romania

**Keywords:** ovarian cancer, HER2 overexpression, phosphatidylcholine-specific phospholipase C, magnetic resonance imaging, magnetic resonance spectroscopy

## Abstract

Antagonizing the oncogenic effects of human epidermal growth factor receptor 2 (HER2) with current anti-HER2 agents has not yet yielded major progress in the treatment of advanced HER2-positive epithelial ovarian cancer (EOC). Using preclinical models to explore alternative molecular mechanisms affecting HER2 overexpression and oncogenicity may lead to new strategies for EOC patient treatment. We previously reported that phosphatidylcholine-specific phospholipase C (PC-PLC) exerts a pivotal role in regulating HER2 overexpression in breast cancer cells. The present study, conducted on two human HER2-overexpressing EOC cell lines - SKOV3 and its *in vivo*-passaged SKOV3.ip cell variant characterized by enhanced *in vivo* tumorigenicity - and on SKOV3.ip xenografts implanted in SCID mice, showed: a) about 2-fold higher PC-PLC and HER2 protein expression levels in SKOV3.ip compared to SKOV3 cells; b) physical association of PC-PLC with HER2 in non-raft domains; c) HER2 internalization and ca. 50% reduction of HER2 mRNA and protein expression levels in SKOV3.ip cells exposed to the PC-PLC inhibitor tricyclodecan-9-yl-potassium xanthate (D609); d) differential effects of D609 and trastuzumab on HER2 protein expression and cell proliferation; e) decreased *in vivo* tumor growth in SKOV3.ip xenografts during *in vivo* treatment with D609; f) potential use of *in vivo* magnetic resonance spectroscopy (MRS) and imaging (MRI) parameters as biomarkers of EOC response to PC-PLC inhibition. Overall, these findings support the view that PC-PLC inhibition may represent an effective means to target the tumorigenic effects of HER2 overexpression in EOC and that *in vivo* MR approaches can efficiently monitor its effects.

## INTRODUCTION

Epithelial ovarian cancer (EOC) is the gynecological malignancy at the highest mortality rate worldwide [1]. A frequently silent progression and late-stage presentation evolve into tumor recurrence and chemoresistance in about two-third of high-grade EOC patients, even after complete response to initial debulking surgery and adjuvant chemotherapy. Molecularly targeted therapies may improve EOC patient management and survival [2].

Antagonizing the aberrant signaling triggered by receptor tyrosine kinases of the human epidermal growth factor receptors family (ErbB) represents a potential strategy for treatment of highly aggressive cancers. In particular, overexpression of the ErbB2 receptor (HER2), first detected in 15%-30% of breast cancers [3], correlates with tumor aggressiveness and poor clinical outcome in a variety of human adenocarcinomas [4]. A deregulated expression of HER2, the preferred dimer partner for the cognate ErbB family members EGFR, HER3 and HER4, is associated with constitutive activation of intracellular signaling pathways responsible for oncogenic cell proliferation and survival [5, 6]. This body of evidence led to the development and approval of anti-HER2 agents for clinical use in patients affected with HER2-overexpressing breast, lung and stomach cancers [7–9].

Although the reported HER2 overexpression rate in EOC patients is highly variable (2%–66%) [10, 11] and its significance as prognostic marker and predictor of patient survival is still controversial [12], the significance of HER2 overexpression as a target for therapy of EOC patients is under active evaluation [13]. Clinical trials utilizing anti-HER2 agents already approved for use in human breast cancers (such as trastuzumab [14], pertuzumab [15] and lapatinib [16]) so far showed only moderate clinical benefit in persistent or recurrent HER2-positive EOC patients [12].

A growing evidence indicates that the aberrant choline phospholipid metabolism of cancer cells can be an effective target for newly designed therapies and allows for the identification of *in vivo* biomarkers of tumor progression and therapy response [17–19]. In this context, we recently showed that phosphatidylcholine (PC)-specific phospholipase C (PC-PLC), enzyme responsible for PC hydrolysis into 1,2-diacylglycerol (DAG) and phosphocholine (PCho) and involved in signal transduction and cell proliferation [18, 20], exerts a pivotal role in regulating HER2 overexpression in human breast cancer cells [21]. In particular, a 66 kDa PC-PLC isoform has been found to accumulate on the plasma membrane of the HER2-overexpressing SKBr3 cell line, where it co-localizes and associates with HER2 in raft domains. PC-PLC inhibition by tricyclodecan-9-yl-potassium xanthate (D609) resulted in HER2 internalization and lysosomal degradation, retarded HER2 re-expression on membrane, reduced HER2 cellular content and anti-proliferative effects [21]. In addition, PC-PLC inhibition was associated with loss of mesenchymal traits in the highly metastatic MDA-MB-231 breast cancer cell line [22].

Exploring in pre-clinical models the molecular mechanisms potentially involved in alternative or combined ways of targeting the HER2-driven oncogenic signaling may foster the development of more effective strategies for treatment of HER2-positive EOC patients. Our previous reports on activation and accumulation on plasma membrane of the 66 kDa PC-PLC isoform in EOC compared with non-tumoral epithelial ovarian cells [23, 24] suggests the interest of investigating the impact of PC-PLC activity on the oncogenic effects of HER-2 overexpression in EOC cells *in vitro* and in xenograft models *in vivo*. To this end, we used two cell-based human EOC models: 1) the at present unique commercially available human HER2-overepressing EOC cell line SKOV3 (HER2^+++^, EGFR^++^, HER3^+/−^, HER4^+/−^, PTEN^+/−^, p53-null) [25] and 2) its *in vivo*-passaged and *in vitro* stabilized SKOV3.ip cell variant. For the latter we previously reported a 1.7-fold higher HER2 protein expression versus SKOV3 cells, associated with 2.9-fold higher PC-PLC activity and enhanced *in vivo* tumorigenicity, as detected by 3-fold faster ascite formation in the peritoneum of SCID mice [26, 27].

With these two HER2-overexpressing EOC cell lines, we investigated the sub-cellular localization of PC-PLC and HER2 and the effects of D609 on PC-PLC inhibition, HER2 mRNA and protein expression, phospho-HER2 (pHER2) and EGFR levels, and cell proliferation. These effects were compared with those induced by trastuzumab on *in vitro* cultured cells. We then evaluated the changes induced by D609 on *in vivo* tumor growth of SKOV3.ip xenografts implanted in immunodeficient mice [28] and evaluated the potential use of functional magnetic resonance (MR) parameters as biomarkers of EOC response to PC-PLC inhibition.

## RESULTS

### Sub-cellular localization of PC-PLC and HER2 in SKOV3.ip compared with SKOV3 cells

**Figure 1:**
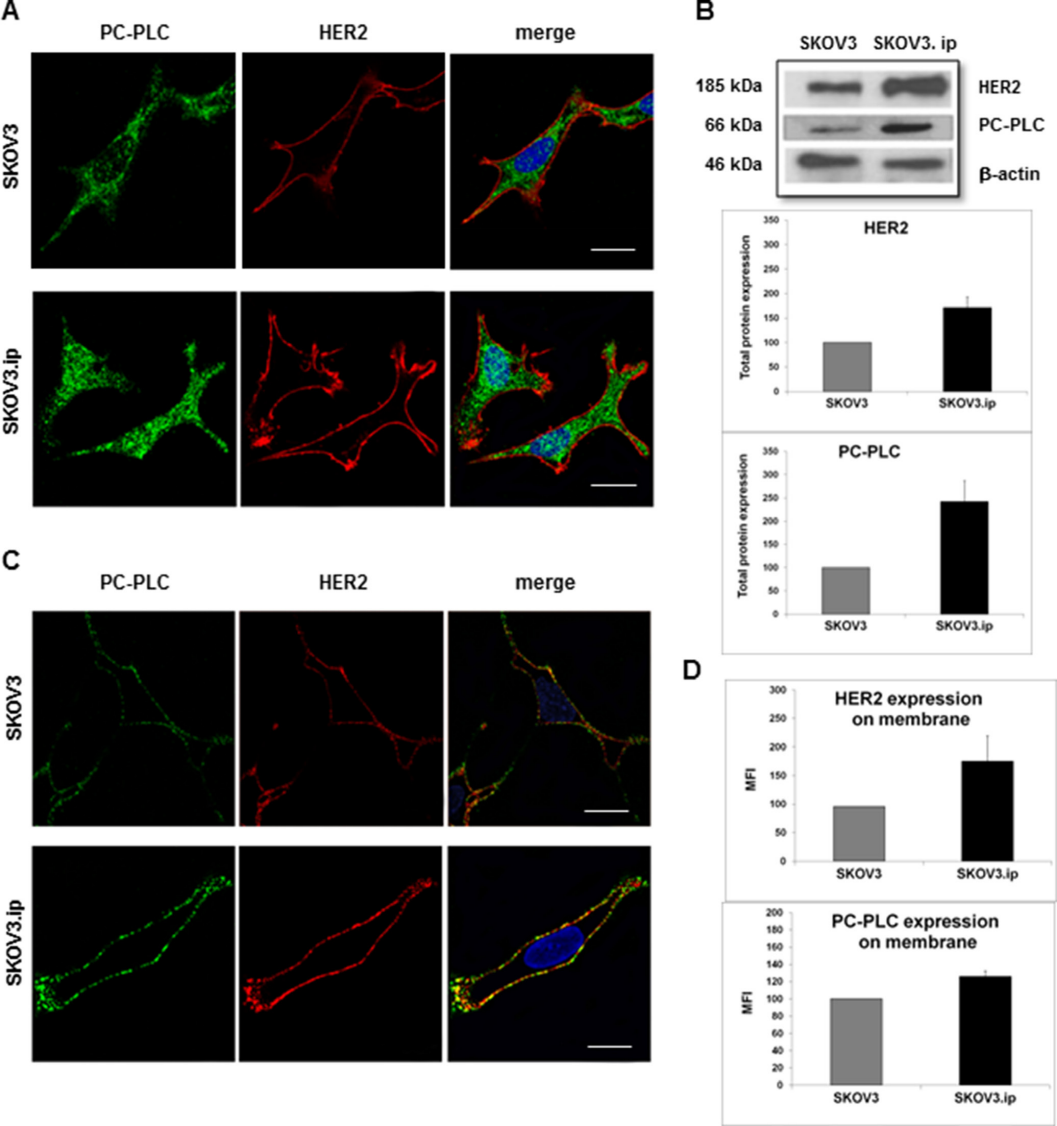
Sub-cellular localization of HER2 and PC-PLC in SKOV3.ip compared with SKOV3 cells (**A**) CLSM analyses (central sections) of fixed and permeabilized cells double-stained with anti-HER2 mAbs (detected in red) and anti-PC-PLC pAbs (green). Nuclei were stained with DAPI (blue). Co-localization of PC-PLC with the nuclear matrix was color-coded in cyan (merge between green and blue). Scale bars, 16 μm for SKOV3.ip; 23 μm for SKOV3; examples of three independent experiments. (**B**) Example of Western blot analysis of total lysate of cells incubated with anti-PC-PLC and anti-HER2 Abs (upper panel); β-actin was used as quantitative loading control. Densitometric analyses (mean value ± SD; *N* = 3) of HER2 (mid panel, *P* = 0.028) and PC-PLC (bottom panel, *P* = 0.032) in SKOV3.ip compared with SKOV3 cells normalized to 100. (**C**) CLSM analyses of unfixed cells (central sections) double stained as in (A). Co-localization between HER2 and PC-PLC on plasma membrane was color-coded in yellow (merge between green and red). Scale bars, 20 μm; examples of three independent experiments. (**D**) Flow cytometry analyses of unfixed SKOV3 and SKOV3.ip cells. The histograms report the mean fluorescence intensity (MFI) values (± SD, *N* = 3) measured for HER2 (upper panel, *P* = 0.049) and PC-PLC (bottom panel, *P* < 0.001).

Confocal laser scanning microscopy (CLSM) of fixed and permeabilized cells showed higher levels of both HER2 and PC-PLC staining in SKOV3.ip versus SKOV3 cells (Figure 1A). Differently from HER2, confined to the cell periphery (left panels), PC-PLC was also present in inner cell compartments in both cell lines (middle panels), including the nucleus. Notably, the presence of PC-PLC-positive granules in the nuclear matrix of these cells (color-coded in cyan in the “merge” panels), particularly evident in the highly invasive cell variant, was in agreement with a previously reported nuclear PC-PLC staining in other cancer cells [22, 29]. Western blot analyses of total cell lysates (Figure 1B) confirmed a 1.7 ± 0.2 (± SD) fold higher HER2 protein level in SKOV3.ip versus SKOV3 cells, as previously reported [27] and showed a 2.4 ± 0.5 fold higher PC-PLC mean protein expression level in the highly tumorigenic cell variant. The higher PC-PLC protein expression was in agreement with the about 3-fold higher activity of this phospholipase previously reported for SKOV3.ip versus SKOV3 cells [27].

CLSM analyses were then performed on unfixed cells in order to obtain more detailed information on PC-PLC localization on plasma membrane in absence of artifacts due to cell fixation and permeabilization [21]. These experiments (examples in Figure 1C) showed higher levels of both HER2 and PC-PLC staining on plasma membrane of SKOV3.ip versus SKOV3 cells. These results were confirmed by flow-cytometry analyses (Figure 1D) which showed a 1.79 ± 0.44 fold increase in the mean fluorescence intensity (MFI) of HER2 in SKOV3.ip versus SKOV3 cells (*P* = 0.049) and a 1.26 ± 0.06 fold increase in that of PC-PLC (*P* < 0.001). CLSM of unfixed cells allowed detection of extensive areas of co-localization of PC-PLC with HER2 on plasma membrane of both cell lines (color-coded in yellow in Figure 1C, “merge” panels).

### Molecular interaction of PC-PLC with HER2 in non-raft domains of SKOV3.ip cells

**Figure 2:**
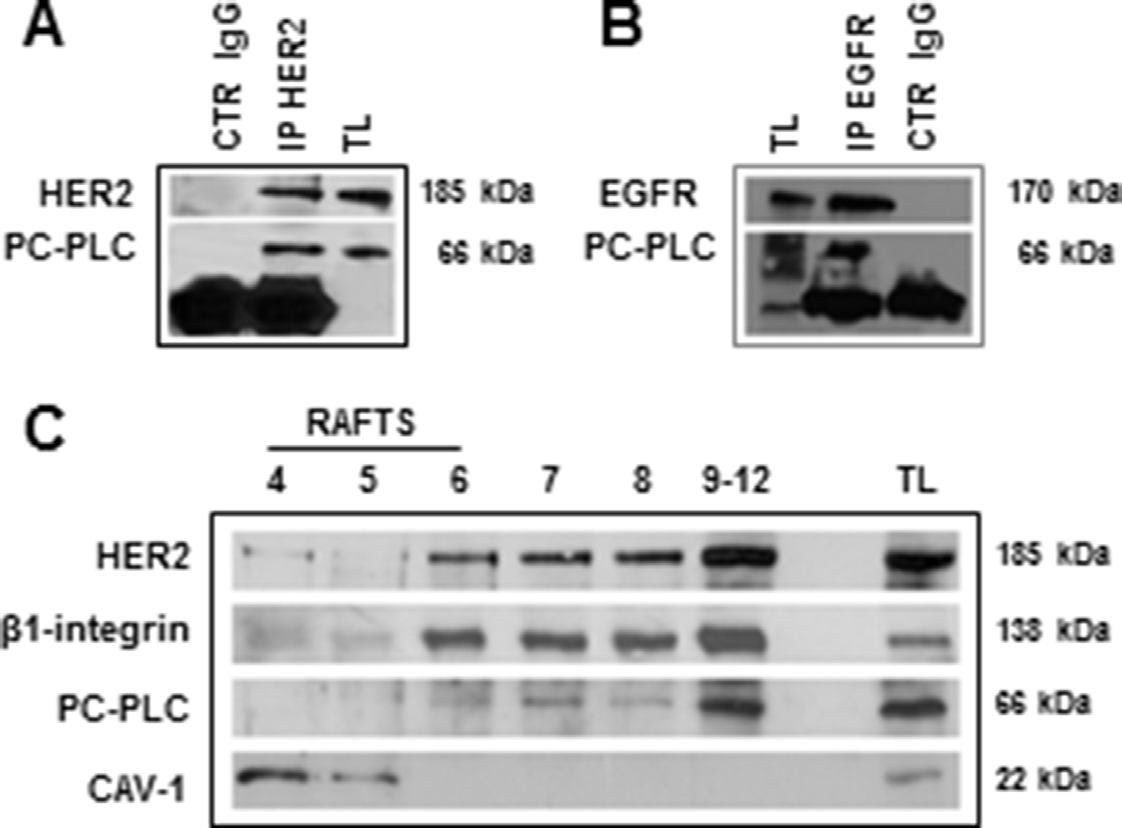
Molecular interaction of PC-PLC with HER2 in non-raft domains of SKOV3.ip cells (**A**, **B**) Western blot analyses of anti-HER2 (A) and anti-EGFR (B) immunoprecipitates (IP), blotted with anti-PC-PLC, anti-HER2 and anti-EGFR pAbs, compared with the respective controls (CTR IgG anti-HER2 and anti-EGFR). (**C**) Sucrose gradient fractions isolated from cell lysates and analyzed by Western blotting for HER2 and PC-PLC detection. TL, total cell lysate. Caveolin 1 (CAV-1) and β1-integrin were used as markers for raft and non-raft domains, respectively.

The co-localization of HER2 with PC-PLC on plasma membrane of the investigated EOC cell lines suggested the possible existence of a physical interaction between the two proteins, as we already reported in HER2-overexpressing breast cancer cells [21]. To investigate this issue we used the SKOV3.ip cell variant, characterized by higher HER2 and PC-PLC protein contents versus the parental cell line. Experiments performed using anti-HER2 Abs and then detecting the precipitated band with anti-PC-PLC and anti-HER2 Abs, showed that the two proteins co-immunoprecipitated (Figure 2A). Analogous experiments showed that PC-PLC also co-immunoprecipitated with EGFR (Figure 2B), suggesting that the phospholipase could form complexes with the two ErbB receptors overexpressed in these cancer cells and/or with HER2/EGFR heterodimers [21].

Western blotting of sucrose-gradient fractions of total SKOV3.ip cell lysates showed the co-existence of PC-PLC and HER2 in non-raft domain fractions characterized by the presence of β1-integrin (Figure 2C). We previously reported that this adhesion protein, known to be involved in metastatic processes, also co-localized with PC-PLC in non-raft domains of a different EOC cell line, OVCAR3 [24]. No substantial amounts of PC-PLC and HER2 were instead detected in raft domains isolated in caveolin-1-containing gradient fractions (Figure 2C).

### HER2 downmodulation in SKOV3.ip cells exposed to the PC-PLC inhibitor D609

**Figure 3:**
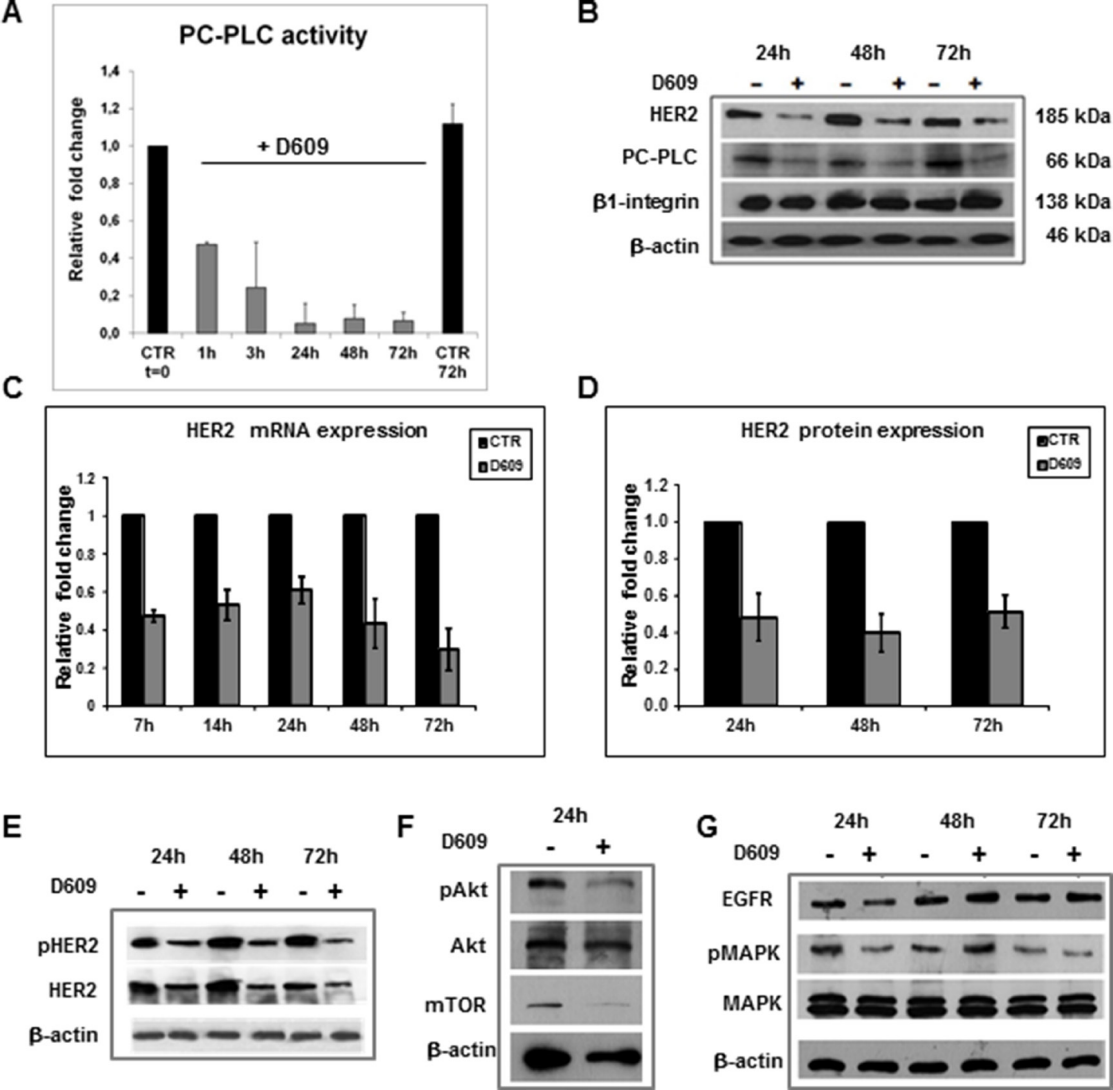
HER2 downmodulation in SKOV3.ip cells exposed to the PC-PLC inhibitor D609 (**A**) Relative changes of PC-PLC activity measured by Amplex Red assays in cells exposed to D609 (50 μg/mL, gray columns) and in untreated controls (CTR at t = 0, normalized to 1.0). The histograms represent mean values ± SD (*N* = 3). (**B**) Example of Western blot analysis of total lysates of cells exposed to D609 compared to untreated controls. The blots were incubated with anti-HER2, anti-PC-PLC and anti-β1-integrin Abs; β-actin was used as quantitative loading control. (**C**) Fold changes of HER2 mRNA levels measured by qRT-PCR (see Methods) in cells exposed to D609 compared with untreated controls (CTR, black columns). The histograms report mean values ± SD (*N* = 3). Statistical significance of differences at each time point: 7 h, *P* = 0.001; 14 h, *P* = 0.008; 24 h, *P* = 0.011; 48 h, *P* = 0.017; 72 h, *P* = 0.009. (**D**) Relative quantification of HER2 protein expression levels (gray columns) in cells exposed to D609 versus the respective untreated controls. The histograms report mean values ± SD (*N* = 6). Statistical significance of differences at each time point versus controls: 24 h, *P* = 0.009; 48 h, *P* = 0.002; 72 h, *P* = 0.002. (**E**–**G**) Western blot analyses as in panel B), incubated with anti-HER2 and anti-phospho-HER2 (pHER2, Tyr1221/Tyr1222) Abs (panel E, example of two independent experiments, +/− D609, 24 h, 48 h, 72 h); or with anti-Akt, anti-pAkt and anti-mTOR (panel F, example of two independent experiments, +/− D609, 24 h); or with anti-MAPK, anti-phospho-MAPK (pMAPK) and anti-EGFR Abs (panel G, example of three independent experiments, +/−D609, 24 h, 48 h, 72 h).

We previously reported a significantly higher PC-PLC activity in SKOV3.ip (13.5 ± 5.8 nmol/10^6^ cells × h) than in SKOV3 cells (4.6 ± 2.0 nmol/10^6^ cells × h) [27]. We therefore selected the SKOV3.ip cell line to investigate the effects of PC-PLC inhibition by D609 on the HER2 expression in EOC. The PC-PLC activity of these cells was already reduced to about 50% of the control value in the first hour of cell exposure to D609 and further decreased to 20% at 3 h and to 5% at 24–72 h (Figure 3A).

Western blot analyses of total lysates of cells exposed to D609 for 24–72 h (example in Figure 3B) showed substantially reduced protein expression levels for both HER2 and PC-PLC, while unaltered levels were detected for β1-integrin.

Quantitative real-time polymerase chain reaction (qRT-PCR) measurements showed that the mean HER2 mRNA expression levels dropped in D609-treated SKOV3.ip cells to 50–60% of the respective untreated controls at 7–24 h and was maintained below 50% at longer times of cell exposure to the PC-PLC inhibitor up to 72 h (Figure 3C). Quantitative Western blot analyses showed that the mean HER2 protein content decreased to 40–50% of the respective control values at 24–72 h of D609 treatment (Figure 3D). These data supported the view that a reduced HER2 gene transcription was likely a major cause for the about 50% HER2 downmodulation detected in D609-treated SKOV3.ip cells.

Regarding proteins present in the downstream signaling pathways, constitutive HER2 phosphorylation was detected by Western blotting in untreated SKOV3.ip cells using an antibody to pTyr 1221/1222 (Figure 3E), in general agreement with an analogous finding reported by Longva et al for SKOV3 cells [30]. Substantial decreases in pHER2 levels occurred in SKOV3.ip cells treated with D609 versus untreated controls (81 ± 3% at 24 h; 52 ± 2% at 48 h and 47 ± 4% at 72 h). Furthermore, while high levels of constitutive Akt phosphorylation (pAkt) were detected in untreated SKOV3.ip cells (Figure 3F), pAkt and mTOR decreased (to 41 ± 1% and 22 ± 13% of the relative controls, respectively) in SKOV3.ip cells exposed for 24 h to D609. Only moderate decreases were instead detected in the mean MAPK phosphorylation levels (84 ± 3% versus untreated controls at 24–72 h; example in Figure 3G). Notably, cell exposure to D609 maintained the EGFR content unaltered (106 ± 16% at 24 h; 96 ± 13% at 48 h; 98 ± 6% at 72 h versus untreated controls; example in Figure 3G). We therefore concluded that of the two ErbB receptors basically overexpressed in these EOC cells, EGFR and HER2, only the latter was significantly reduced following cell exposure to the PC-PLC inhibitor.

### Internalization of HER2 and PC-PLC re-localization in SKOV3.ip cells exposed to D609

**Figure 4:**
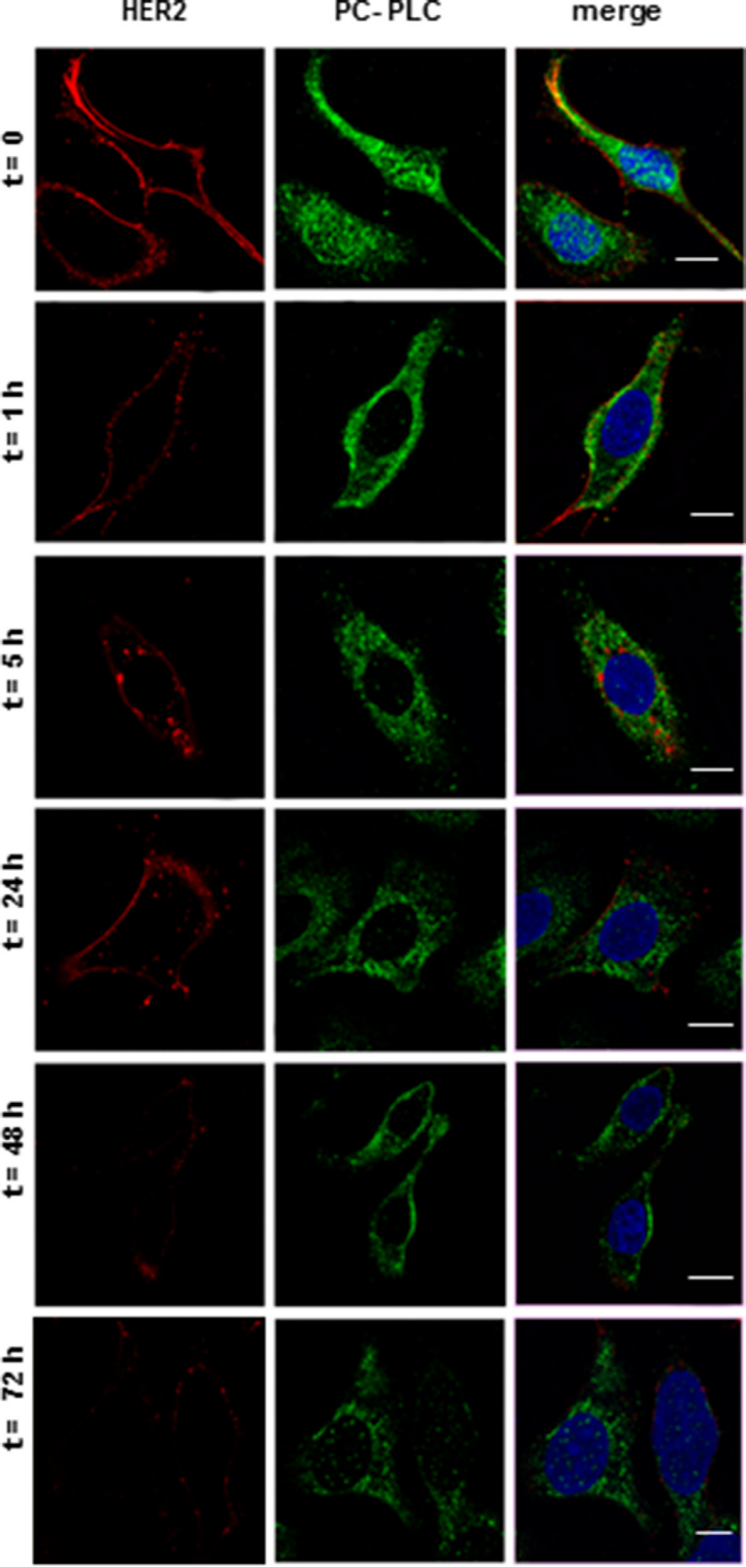
Internalization of HER2 and PC-PLC re-localization in SKOV3.ip cells exposed to D609 CLSM analyses (central sections) of cells cultured either in absence (t = 0) or presence of D609 for the indicated time intervals. After washing, cells were fixed and stained with anti-HER2 mAb (detected in red) or anti-PC-PLC pAbs (green). Nuclei were stained with DAPI (blue). Co-localization between PC-PLC and the nuclear matrix was color-coded in cyan (merge between green and blue). Scale bars, 15 μm. Panels show representative examples of five independent series of experiments.

CLSM analyses of SKOV3.ip cells harvested and fixed at different times of exposure to D609 (examples in Figure 4) already showed the appearance of HER2-positive structures in inner cell compartments at 5 h of D609-treatment, while the peripheral HER2 staining progressively decreased, consistently with endocytosis and retarded re-expression of the receptor on membrane, as already reported for D609-treated SKBr3 cells [21]. Overall, data reported in Figure 3 and Figure 4 support the view that the D609-induced HER2 downmodulation derives from the combination of at least two molecular mechanisms, i.e. a reduction of HER2 gene transcription and degradation of the internalized HER2 protein overexpressed on plasma membrane.

Regarding changes induced by D609 in the PC-PLC sub-cellular localization, the most striking effect was the rapid loss (within one hour) of the PC-PLC positive granules located in the nuclear matrix. The molecular mechanisms responsible for this phenomenon need future investigations to elucidate the role of nuclear PC-PLC as a possible co-factor in gene transcription, perhaps through NF-kB activation [31, 32].

### Differential effects of D609 and trastuzumab on HER2 protein expression, cell proliferation and cell cycle in SKOV3.ip cells

**Figure 5:**
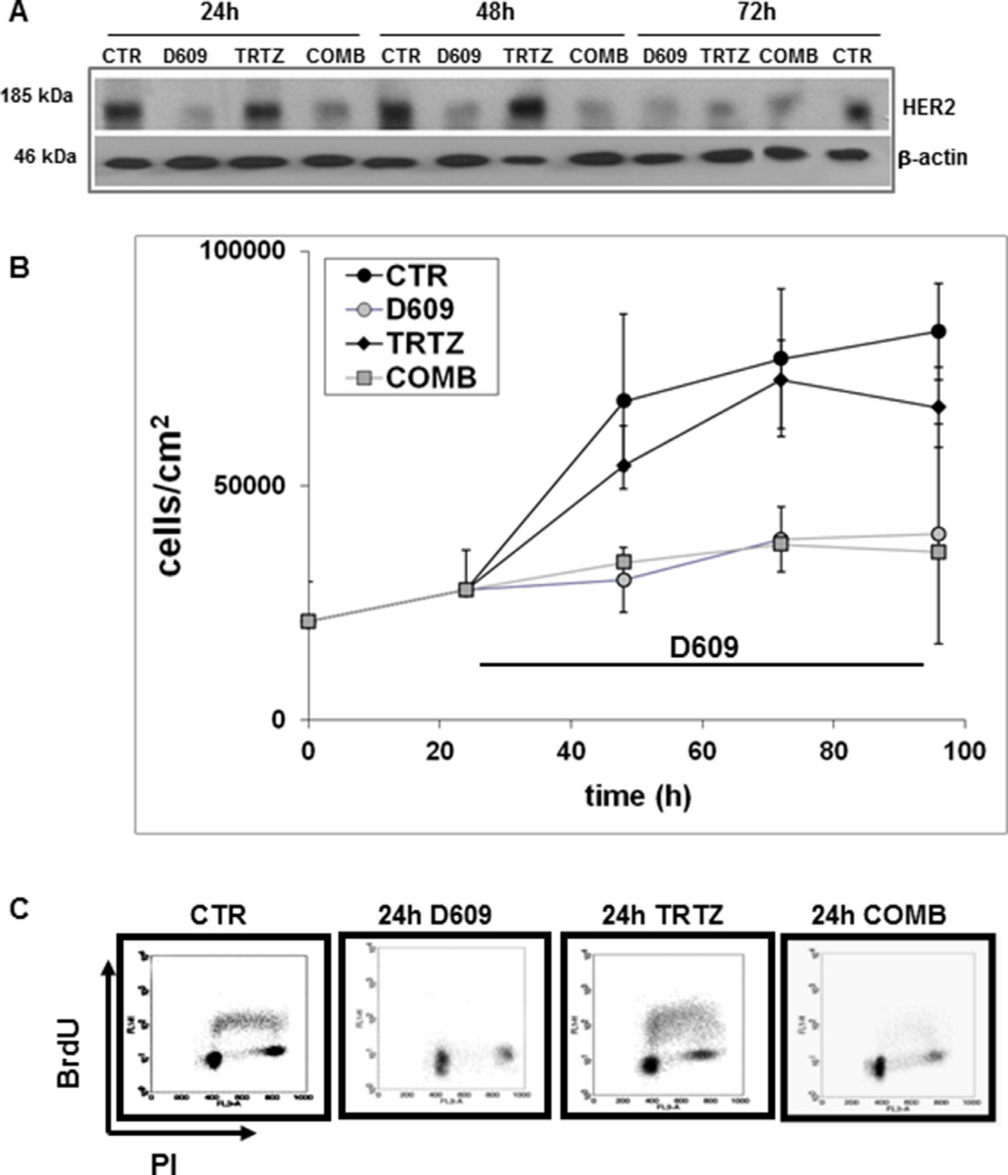
Differential effects of D609 and trastuzumab on HER2 protein expression, cell proliferation and cell cycle of SKOV3.ip cells (**A**) Changes in the overall HER2 protein expression detected by anti-HER2 pAbs in Western blot experiments of total lysates of cells following exposure for different time intervals to D609 (50 μg/mL), trastuzumab (TRTZ, 10 μg/mL) or their combination (COMB). CTR, untreated control cells; example of three independent experiments. (**B**) Cell growth curves (mean values of cell counts ± STD, *N* = 3) of cells seeded at *t* = 0, cultured for 24 h and then treated for different times (24, 48 and 72 h) with either TRTZ, D609 or COMB, or left untreated (CTR). Statistical significance of differences: P(D609 versus CTR) = 0.030 at 24 h; 0.024 at 48 h; 0.010 at 72 h; P(TRTZ versus CTR) = 0.270 at 24 h; 0.690 at 48 h; 0.040 at 72 h. Differences between D609- and COMB-treated cells were not significant. (**C**) Flow cytometry analyses of cells performed under the same conditions as in panels (A) and (B) following 24 h of cell exposure to the different agents compared with the respective controls. Representative examples of dot plots showing DNA content (x-axis, propidium iodide (PI) fluorescence) and bromodeoxyuridine (BrdU) incorporation (y-axis, log FITC fluorescence) are reported.

Western blot experiments (example in Figure 5A) were conducted to compare the effects induced on the overall HER2 protein content by cell exposure to either D609, trastuzumab (TRTZ, 10 μg/mL) or their combination (COMB). Differently from D609-treated cells, for which significant 50%–60% decreases were confirmed in HER2 expression at 24–72 h (in agreement with data reported in Figure 3D), cells exposed to TRTZ maintained a practically unaltered HER2 level up to 48 h (*P* = 0.418) and showed only a borderline significant decrease to 60% at 72 h (*P* = 0.066). Densitometric analyses of three independent series of Western blot experiments showed that the effects of D609 and COMB were not significantly different (P (D609 versus COMB) = 0.354 at 24 h, 0.527 at 48 h and 0.640 at 72 h).

Significant antiproliferative effects were detected in SKOV3.ip cells continuously exposed for 24–72 h to D609, while treatment with TRTZ resulted in a decrease of cell count only at 72 h (Figure 5B). No significant differences were found in the proliferation of cells exposed to either D609 or COMB. Furthermore, flow-cytometry analyses showed that, following 24-hour exposure to D609, cells underwent cell cycle arrest in G0-G1, while the S phase fraction dropped to 8% (Figure 5C). A similar effect, with a drop of the S phase to 5%, was observed with the combination of D609 and TRTZ (Figure 5C), without induction of any detectable sub-G0 peak. Continuous exposure to TRTZ alone maintained the mean percentage of the S-phase fraction close to that of untreated controls (35%).

### Effects of D609 on *in vivo* tumor growth and MR parameters of SKOV3.ip xenografts in SCID mice

**Figure 6:**
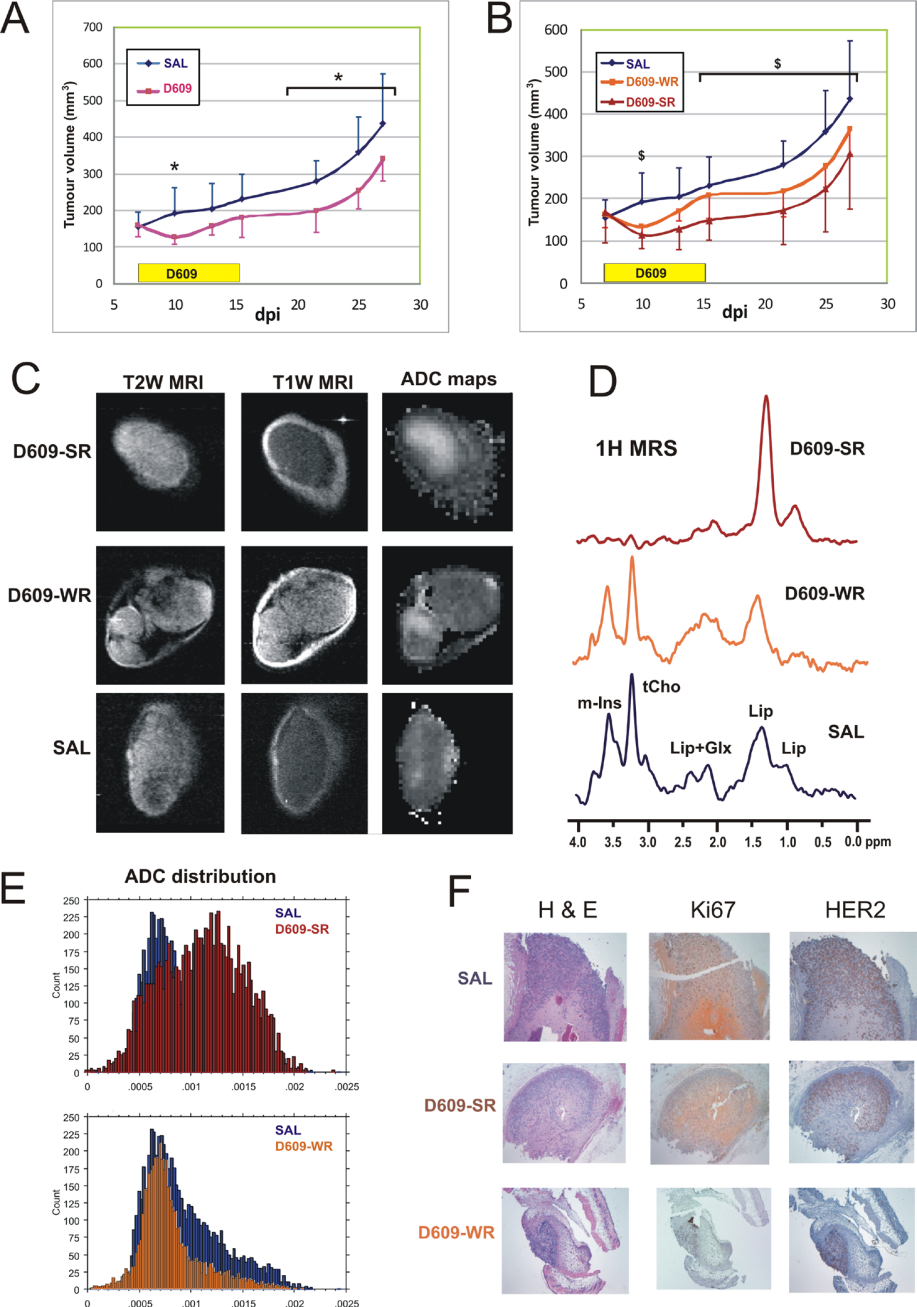
Effects of D609 on *in vivo* growth and MR parameters of SKOV3.ip xenografts in SCID mice (**A**) Tumor growth of SKOV3.ip xenografts and effects of 9 daily consecutive administrations of D609 (1 mg/mouse/day between day 7 and day 15) on tumor volume. *P < 0.05, volume of D609-treated versus control tumors (SAL). (**B**) Tumor growth of the same xenografts as in panel (A), where D609-treated tumors have been divided into two subgroups according to their different level of sensitivity to treatment (D609-SR and D609-WR) evaluated on the basis of tCho content and ADC values. ^$^*P* < 0.05, volume of D609-SR treated versus control (SAL). (**C**) Representative examples of coronal T2- and T1-weighted MR images and ADC maps. The coronal orientation was chosen in order to avoid mouse body within the images and therefore obtain higher in plane resolution (60 × 120 μm). (**D**) ^1^H MR spectra acquired *in vivo* from D609- and saline (SAL)-treated xenografts (PRESS, TR/TE = 4000/23 ms). Peak assignment: mIns, myo-inositol; tCho, choline-containing compounds; Lip, lipids; Glx, glutamine plus glutamate. (**E**) ADC distributions of *in vivo* D609-treated xenografts (D609-SR and D609-WR) are compared with the controls (normalized to the control values). A marked increase in the percentage of the tumor exhibiting high ADC values was observed for the D609-SR group. (**F**) Histological characterization by haematoxylin and eosin (H&E) staining (left panels) and immunohistochemical characterization by antigen proliferation index Ki67 (middle panels) and HER2 expression (right panels) of *ex vivo* tumor xenograft samples dissected after 9 doses of saline (SAL)- or D609-treatments (D609-SR, strong responder to D609 treatment; D609-WR, weak responder to D609 treatment).

*In vivo* intraperitoneal (ip) treatment with D609 induced significant decreases (*P* = 0.02) in the volume of SKOV3.ip xenografts implanted in female SCID mice compared with controls treated with saline solution (SAL) (Figure 6A). In particular, a 30% reduction of the mean tumor volume occurred as early as 72 h after the first D609 administration, this difference being practically maintained between 20% and 30% up to 12 days after the last D609 injection (see Supplementary Results). Measurements of *in vivo* MRS and functional MRI parameters allowed separation of the D609-treated xenografts in two distinct subgroups: “stronger responders”, D609-SR (*N* = 6) and “weaker responders”, D609-WR (N = 7) (Table 1 and Figure 6B–6F). The D609-SR group was identified by a tCho level below detection, apparent diffusion coefficient (ADC) larger than 11 × 10^–4^ mm^2^/s and increases in the mean T2 and perfusion values, associated with a stronger volume growth rate reduction. The D609-WR group was characterized by tCho, ADC, T2 and perfusion values comparable to those of SAL-treated tumors, and showed a weaker volume growth rate reduction, as described in detail below.

**Table 1:** Quantification of tumor specific growth rate (calculated after the first three doses of D609-treatment) and MRS/MRI parameters (measured between day 11 and 15 post-implantation, during treatment) in D609-SR, D609-WR and SALINE (SAL)-treated xenograft groups

	SAL (*n* = 10)	D609-SR (*n* = 6)	D609-WR (*n* = 7)	F *, *P*
tCho (mM)	4.4 ± 1.9	b. d.	4.8 ± 1.7 ^+^	F (2, 20) = 15.377 *P* < 0.0001
ADC (10^-4^mm^2^/s)	8.2 ± 1.5	12.1 ± 0.9^§^	7.5 ± 0.9 ^+^	F (2, 20) = 24.699 *P* < 0.0001
Skewness	0.9 ± 0.6	−0.2 ± 0.4^§^	0.8 ± 0.6 ^+^	F (2, 20) = 7.786 *P* = 0.003
VSF (%)	5 ± 2	7 ± 1^§^	5 ± 1	F (2, 20) = 3.875 *P* = 0.04
T2 (ms)	73 ± 18	92 ± 37	67 ± 24	n.s.
Specific growth rate (d^-1^)	0.07	−0.13^§^	−0.06	F (2, 17) = 3.726 *P* = 0.046

*ANOVA test; ^§^*P* < 0.05, D609-SR versus control tumors (SAL); ^+^*P* < 0.05, D609-SR versus D609-WR. D609-SR, strong responder to D609 treatment; D609-WR, weak responder to D609 treatment; b.d., below detection; d, day.

Significant differences were detected between the mean volumes of SAL and D609-SR tumors at all time points (except 13 dpi), but not between SAL and D609-WR, nor between D609-WR and D609-SR subgroups (Figure 6B). The specific tumor volume growth rates [33] were markedly different at the start of treatment (being 0.07, -0.06 and -0.13 per day for SAL, D609-WR and D609-SR, respectively, between day 7 and day 10 of tumor growth) showing a volume shrinkage of the D609-treated tumors, more evident for the D609-SR tumors. This initial tumor shrinkage caused a growth reduction for the D609-treated groups still evident up to the end of the experiment (with specific volume growth rates 0.05, 0.04 and 0.03 per day for SAL, D609-WR and D609-SR, respectively, between day 7 and day 27 of growth).

Measurements of *in vivo* metabolic and functional MR parameters (Table 1; Figure 6C–6F) showed significant differences among the three groups. Figure 6C reports representative examples of the different features of T2-weighted (T2W), T1-weighted (T1W) images and apparent diffusion coefficient (ADC) maps obtained for the three groups. Table 1 shows that these groups differed in tCho content (*P* < 0.0001) (example in Figure 6D), ADC mean value (*P* < 0.0001) (example in Figure 6E), vascular signal fraction (VSF) mean value (percentage of perfusion, *P* = 0.04), and mean value of skewness (i.e. the asymmetry with respect to the normal distribution of ADC values within the tumors, *P* = 0.003). Bonferroni posthoc comparisons showed significant differences between the mean tCho, ADC and skewness values of D609-SR versus controls, and D609-SR versus D609-WR tumors. The VSF values showed significant difference only between D609-SR and SAL-treated tumors (Table 1). Differences could also be observed in the ADC distribution (Figure 6E) where D609-SR showed a broader distribution centered at higher ADC values when compared with SAL and D609-WR tumors. Differences in skewness could also be observed. Notably, the histograms of controls and D609-WR tumors (Figure 6E, lower panel) had a prominent peak (viable part of the tumor) associated with a right wing (positive skewness) which could be attributed to necrotic areas [34]. The histograms of D609-SR tumors (Figure 6E, upper panel) showed instead a broader peak shifted to higher ADC values, which was more symmetric and was associated with a small left wing (negative skewness) likely due to still viable tumor areas (packaged cells). No differences were instead observed in the kurtosis of the three groups, which showed a similar dispersion of ADC values from the mean.

The marked changes observed in MRS and MRI parameters were associated in SKOV3.ip xenografts with an average 57% reduction of the proliferation index Ki67 and decreased (from strong to moderate/weak) HER2 staining in tumors dissected during D609 treatment. Examples of histopathological analyses of dissected D609-SR, D609-WR and SAL-treated tumors are shown in Figure 6F. The Ki67 and HER2 staining levels were then restored to control values in tumors dissected 12 days after the end of the overall treatment (data not shown).

**Figure 7:**
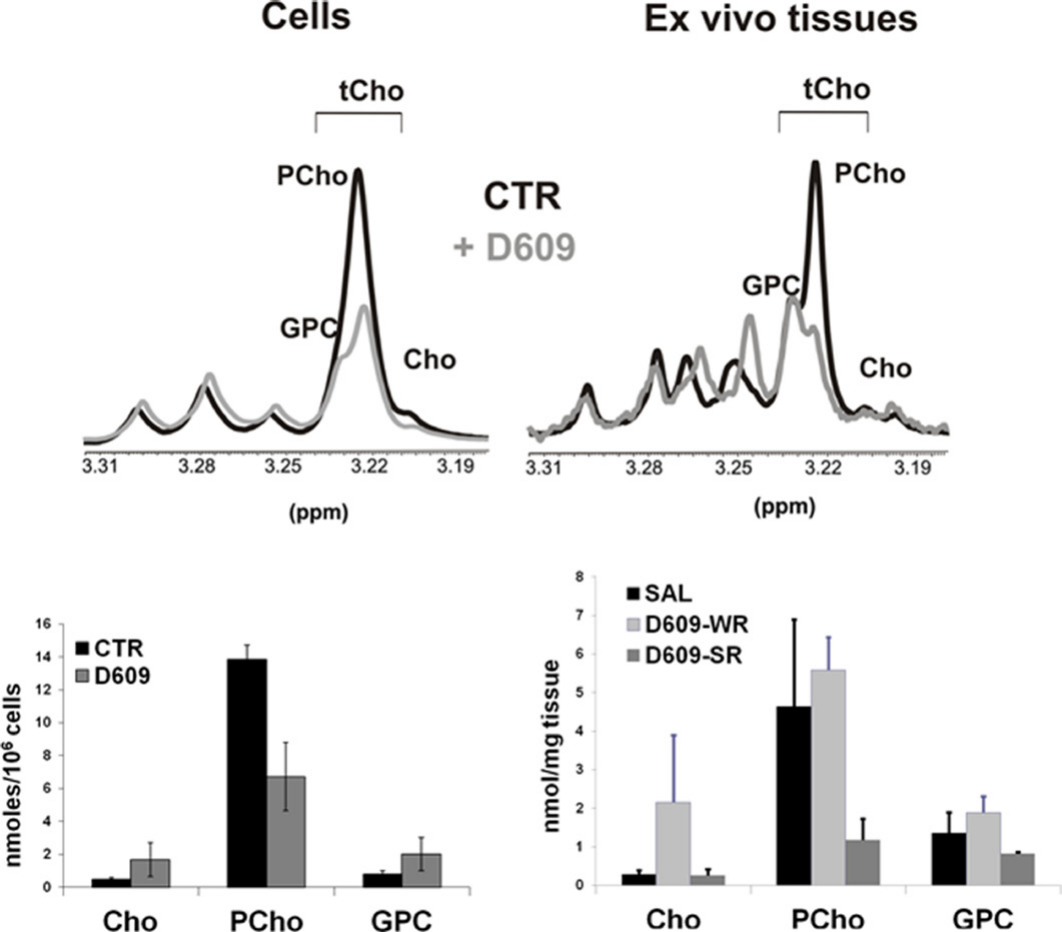
Changes induced on the total choline (tCho) metabolic profile of *in vitro* SKOV3.ip cells and their *in vivo* xenografts, upon treatment with D609 Upper panels: representative partial ^1^H NMR (9.4 T) spectra of aqueous extracts of untreated (CTR) and D609-treated cells (left) and specimens dissected from D609-strong responder (D609-SR) and saline (SAL)-treated SKOV3.ip xenografts (right). *Peak assignments*: Cho: free choline; GPC: glycerophosphocholine; tCho, total choline-containing metabolites; PCho, phosphocholine. Lower panels: absolute quantification of choline-containing metabolites in aqueous extracts of untreated (CTR) and D609-treated cells (left) and in aqueous extracts of tissue specimens dissected from D609-SR, D609-WR and SAL-treated SKOV3.ip xenografts (right). D609-SR, strong responders to D609 treatment; D609-WR, weak responders to D609 treatment.

Quantitative MRS analyses of extracts of tumor tissues dissected during ip treatment with D609 showed a 41% reduction of PCho/tCho ratio in D609-SR tumors compared with SAL-treated controls (*P* = 0.006, *N* = 3, Figure 7, right panels). A similar decrease in intracellular PCho content was detected in SKOV3.ip cells (Figure 7, left panels) following 24-hour *in vitro* exposure to D609.

Preliminary experiments in which D609 was locally injected peritumorally showed clearcut decreases in Ki67 staining of tumor specimens dissected four days after the end of treatment (Supplementary Figure 1). These data suggested that an efficient access of D609 to the tumoral area may be relevant to its effects on *in vivo* tumor growth.

## DISCUSSION

Here we report that PC-PLC, a C-type phospholipase specific for the major phospholipid of eukaryotic cells, co-localizes with HER2 in the HER2-overexpressing SKOV3 cell line and in its highly *in vivo* tumorigenic SKOV3.ip cell variant, characterized by a 2-fold higher HER2 overexpression and a significantly higher mean PC-PLC activity [27]. These features were associated with a faster *in vivo* formation of ascites in mice transplanted with SKOV3.ip cells, while the *in vitro* cell proliferation was similarly high in both cell lines [27]. This evidence suggests that a higher level of HER2 overexpression and PC-PLC activation may have a role in maintaining a higher level of *in vivo* tumor growth, likely by allowing these cancer cells to better coping with the hostile *in vivo* environment.

Co-immunoprecipitation tests showed that PC-PLC forms complexes with both HER2 and EGFR receptors, as previously reported for HER2-overexpressing SKBr3 breast cancer cells [21]. Long-term cell exposure of SKOV3.ip cells to D609 resulted in a) PC-PLC activity inhibition (down to 20% of untreated controls at 3 h and to less than 5% at 24 h); b) HER2 internalization (already detected at 5 h); c) decrease in HER2 mRNA expression (already detected at 7 h); d) decreased HER2 (but not EGFR) protein expression at 24–72 h; e) lower pHER2 content at 24–72 h; f) decreased pAkt and mTor levels at 24 h and g) long-standing cell cycle arrest.

The lack of a parallel EGFR downmodulation in D609-treated SKOV3.ip cells indicated that the effects of PC-PLC inhibition were selectively targeted against HER2, out of the two ErbB receptors overexpressed in these cells. The parallel decrease in PC-PLC protein content in the investigated D609-treated SKOV3.ip cells was not a general effect of this inhibitor in cancer cells, since no similar changes were found in the mean PC-PLC protein level in the HER2-overexpressing SKBr3 cells exposed to D609. This variability may depend upon different mechanisms of ubiquitination and degradation of complexes formed by PC-PLC with membrane receptor in different cancer cell types.

The changes induced by D609 on HER2 and pHER2 contents and on cell proliferation of SKOV3.ip cells were markedly different from the effects reported in the literature for SKOV3 cells treated with trastuzumab. In particular, the humanized anti-HER2 monoclonal antibody did not induce endocytic HER2 downregulation, did not alter the pHER2 levels, and did not affect the *in vitro* proliferation of SKOV3 cells [30]. The lack in these cells of functional PTEN (phosphatase and tensin homologue) was held responsible by Longva and coll [30] for the absence of an effective trastuzumab-mediated inhibition of the PI3K/Akt signaling pathway, with the consequent maintenance of high levels of constitutive Akt phosphorylation.

These results warrant further investigations on the effects of PC-PLC inhibition on post-receptor signaling pathways responsible for EOC cell differentiation and proliferation. It is in fact known that the formation of HER2 heterodimers with cognate members of the ErbB2 family activates and prolongs a rich signaling network including MAPK and PI3K/Akt pathways and the downstream reaction cascades which induce distinct transcriptional programs in the nucleus [6]. Agents affecting HER2 expression and mechanisms of HER2 endocytosis and recycling may therefore have profound effects on HER2-driven oncogenic signaling transduction [12]. Although detailed investigations on the effects of D609 on HER2-driven oncogenic signaling pathways were out of the scope of the present study, our Western blot experiments confirmed high levels of constitutive Akt phosphorylation in untreated SKOV3.ip cells, and showed decreases in the levels of pAKT and mTOR in cells exposed to D609 for 24 h. Interestingly, we recently reported that PC-PLC inhibition by D609 also induced downmodulation of the CXCR4 receptor in U87MG glioma cells, an effect which was similarly associated with remarkable decreases in AKT phosphorylation and reduced cell proliferation [35]. The MAPK phosphorylation levels were instead maintained rather high in D609-treated SKOV3.ip cells (84 ± 3% versus untreated controls at 24–72 h), likely due to the reported constitutive Ras activation present in these cells [30]. In conclusion, although further investigations are needed to elucidate the effects of D609 on signaling pathways downstream of HER2 activation, our present results suggest that PC-PLC inhibition can represent a new potential strategy to counteract the oncogenic effects of HER2 amplification in HER2-overepressing EOC cells. On the other hand, the maintenance of high EGFR levels in D609-treated SKOV3 cells suggests the interest of preclinically evaluating the effectiveness of appropriate combinations of a PC-PLC inhibitor with specific anti-EGFR agents.

Elucidation of the molecular mechanisms responsible for the observed dependence of HER2 expression upon PC-PLC activity may be relevant to guide the design of potential new targeted therapies. We hypothesize that the molecular interaction of HER2 with an activated PC-PLC isoform may play a key role on the expression and function of this receptor, by contributing to generate around it, in combination with other activated PC-cycle enzymes (e.g. choline kinase alpha and phospholipase D) a local accumulation of PC precursors and lipid second messengers such as PCho, DAG and phosphatidate, needed to convey the HER2 oncogenicity into cell growth and proliferation [36, 37]. Furthermore a local overproduction of DAG generated by PC-PLC-mediated hydrolysis could also play a structural role on HER2 homing into specialized membrane domains. An anomalous local concentration of DAG has in fact been shown to perturb the phospholipid bilayer, to alter protein-lipid interactions and influence the formation of membrane microdomains, thus modifying the exposure of surface membrane receptors and affecting their recycling between membrane and inner cell compartments [38, 39]. Further investigations are needed to elucidate the mechanisms whereby HER2 is located in non-raft domains in the investigated EOC cells, instead of being located in raft domains, as detected in HER2-overexpressing SKBr3 breast cancer cells [21]. However, it appears worth-noting that in both cancer cell types PC-PLC is physically associated with HER2 and its enzymatic inhibition leads to a substantial downmodulation of this receptor. The proposed interpretation of the role played by this physical association in EOC is also supported by a) the parallel increase of both PC-PLC activity and HER2 overexpression in SKOV3.ip versus those of the less tumorigenic parental SKOV3 cells [27] and b) the strong decrease of PCho (co-produced with DAG via PLC-mediated PC hydrolysis) in both *in vitro* cultured SKOV3.ip cells and *in vivo* SKOV3.ip xenografts, following treatment with D609 (Figure 7). According to the proposed mechanisms, the presence of an activated PC-PLC isoform in HER2-overexpressing EOC cells might therefore be seen as a sort of “chaperone” that helps the HER2 receptor to be anchored to plasma membrane domains, while the HER2 oncogenic action can be partly switched off through cell exposure to a PC-PLC inhibitor.

The detection of PC-PLC-positive granules in various cell compartments of HER2-overexpressing EOC cells (membrane, cytoplasm and nuclear matrix) indicates that this enzyme is involved in multiple cell functions, including regulation of cell cycle and cell proliferation, in agreement with previous observations on PDGF-stimulated compared with quiescent fibroblasts [40] and highly proliferating squamous carcinoma cells (A431) compared with non-tumoral keratinocytes [29]. The activation of PC-PLC in the nucleus could also produce DAG, via a nuclear PC cycle, thus leading to a long term response such as mitosis through the activation of protein kinase C [41]. The rapid changes of nuclear PC-PLC content in D609-treated cancer cells may have a still poorly investigated role as a co-factor in transcription [31, 32].

Overall, these data and their proposed interpretation suggest that PC-PLC activation is relevant to sustaining the post-receptor signaling activated by HER2 overexpression in EOC cells.

The enhanced *in vivo* tumorigenicity of the SKOV3.ip cell variant allowed us to combine *in vitro* with *in vivo* investigations on the effects of D609 on a highly aggressive HER2-overexpressing EOC model, using both *in vitro* cultured cells and their xenografts implanted in immunodeficient mice. Decreases in tumor growth, as well as in HER2 and tCho (mainly PCho) contents were observed in SKOV3.ip xenografts during *in vivo* D609-treatment administered through ip injections. These experiments clearly showed an early reduction of tumor growth soon after the start of treatment, associated with decreases in Ki67 antigen labeling index and in HER2 staining in specimens dissected from mice euthanized during the treatment schedule.

Previous studies reported that substantial therapeutic effects of D609 were associated with low toxicity profiles in preclinical *in vivo* models. In particular, a human non-small cell lung carcinoma xenograft showed extensive tumor necrosis after intravenous injection of D609 (up to 2 mg/mouse) [42, 43], while inhibition of PC-PLC activity was obtained in human prostate carcinoma xenografts at a lower D609 dose (1 mg/mouse) [44]. Our experiments confirmed the lack of toxic side-effects in mice treated with D609 at 1 mg/mouse (see Supplementary Results).

In our model, following *in vivo* D609 treatment, we identified two equally balanced subgroups of tumors with different levels of metabolic and functional parameters (D609-WR and D609-SR). A dual response of molecular imaging biomarkers has already been reported for different types of targeted anticancer therapies applied to preclinical models [45]. There is an intrinsic variability in every model, due to different factors (site of injection, inflammation, different accessibility of the drug during tumor growth because of different levels of tumor vascularization, etc). Interestingly, in our experiments the D609-SR tumors had the highest VSF (as shown in Table 1), which corresponded to a higher vascularization and therefore higher probability for a diffuse drug delivery within the entire tumor mass. So we could conclude, in agreement with preliminary experiments performed with peritumoral D609 administration, that an effective accessibility of D609 molecules to the overall tumoral area could be relevant to the effects of this inhibitor on tumor growth and metabolism. It seems therefore crucial to identify and monitor possible subgroups of tumors with enhanced response to a newly proposed molecular treatment.

Our previous preclinical studies showed the capability of DWI to detect ovarian masses developed in SCID mice following subcutaneous or intraperitoneal implantation of tumor cells [26] and monitor early therapy response to an anti-angiogenic or platinum-based treatment of human EOC models [27, 46]. On the other hand, clinical studies showed that DWI (along with measures of the ADC mean value and distribution) may be the most appropriate imaging modality for monitoring [47, 48] or predicting [49] the response to chemotherapy of patients with advanced EOC. DWI can therefore be successfully used in EOC patients for disease localization and monitoring of treatment response and recurrence [50, 51].

Here, we observed a marked ADC increase in our EOC model during D609 treatment, corresponding to an increase in the intratumoral extracellular space, in agreement with microscopic or macroscopic areas of necrosis, as already confirmed by histology in previous works [26, 27, 34, 46], possibly due to hypoxia or to destruction of cellular membranes following the anticancer drug treatment. The broad ADC distribution is an indicator of high heterogeneity in the tumor response, which is typically higher in the central part of the tumor. Furthermore, we detected significant differences between D609-SR and SAL-treated tumors, in the VSF parameter as an index of vascular component. We can attribute the increase in VSF to macroscopic necrosis in the central part of the tumors which also showed a reduced tCho signal.

Overall, the use of integrated MRI approaches which include ADC (mean values and distribution) and MRS may be crucial to the assessment of early tumor response to a targeted therapy including PC-PLC inhibition, because of the capability of these approaches to detect alterations at cellular and physiological level (beyond the classical response evaluation criteria in solid tumors (RECIST)).

This study, despite some limitations, opens the way to new research areas. First, D609 is currently the only commercially available PC-PLC inhibitor, supporting the needs of developing and testing novel selective PC-PLC inhibitors. Second, the effects of D609 could be tested only in two preclinical *in vitro* and *in vivo* models. However, the use of the at present unique HER2-overepressing EOC cell line SKOV3 and its *in vivo*-passaged and stabilized SKOV3.ip cell variant provided peculiar advantages for evaluating the impact of PC-PLC on HER2 overexpression and oncogenicity. Thus, the results of this study suggest the interest of further exploring the role of inhibiting PC-PLC to weaken the HER2-mediated oncogenic signaling in primary cell cultures derived from EOC patients, as a first step towards the construction of personalized therapeutic protocols.

Overall our findings may foster the development of more effective strategies for treatment of HER2-positive EOC patients.

## MATERIALS AND METHODS

### Cells, antibodies and reagents

The human HER2 and EGFR overexpressing ovarian serous cancer cell line of ascitic origin SKOV3 was purchased from ATCC, Manassas, VA. The SKOV3.ip cell line was established in our laboratory from ascitic exudates produced in female SCID mice following ip injection of SKOV3 cells [27]. Both the utilized cell lines were subjected to short tandem repeat (STR) analysis in accordance with the ATCC guidelines, and authenticity was confirmed. Details on preparation and characterization of polyclonal antibodies (pAbs) recognizing a mammalian PC-PLC isoform (66 kDa) [40, 52, 53]; pAbs and/or monoclonal antibodies (mAbs) against HER2, phospho-HER2 (pHER2), EGFR, β1-integrin, caveolin-1, MAPK, phospho-MAPK (pMAPK), Akt, phospho-Akt (pAkt), mTOR; details on reagents for immunofluorescence analyses, biochemicals and chemicals; and further details on experimental procedures are reported in Supplementary Materials.

### Treatment of *in vitro* cultured cells

The procedures for treatment of cells with D609, trastuzumab or their combination and subsequent cell-based assays are reported in Supplementary Methods.

### PC-PLC activity assay

The PC-PLC activity was determined in total cell lysates using an Amplex Red^®^ PC-PLC-specific assay kit (Molecular Probes) according to the manufacturer's protocol modified as described [24].

### Western blot analyses and co-immunoprecipitation tests

Protein expression was evaluated by densitometric analysis of Western blot experiments performed on total cell lysates (20 μg of proteins) as described [24]. β-actin was used as quantitative loading control.

Total cell lysates (1 mg/mL) were incubated with 10% protein G Sepharose (Amersham Biosciences) and with either anti-HER2 (Santa Cruz Biotechnology) or anti-EGFR specific Abs overnight at 4°C. Immunoprecipitates were washed, denatured, resolved by 7% SDS-PAGE under reducing conditions and blotted with the related Abs, to detect the presence of PC-PLC, HER2 and EGFR in the co-immunoprecipitated complexes, as described [21].

### Separation of lipid raft and non-raft fractions by sucrose gradient

Cell growth, gradient fractionation of cell lysates, protein separation and detection of the distribution of HER2 and PC-PLC in the gradient fractions were performed as reported [21, 24, 54] and described in detail in Supplementary Methods.

### CLSM and flow-cytometry analyses

CLSM observations were performed with a Leica TCS SP2 AOBS apparatus, using a 63x/1.40 NA oil objective and excitation spectral laser lines at 405, 488 and 594 nm. Image acquisition and processing were carried out using the Leica Confocal Software 2.6 rel 1537 (Leica, Wetzlar, Germany) and Adobe Photoshop CS2 1.4.10 software programs (Adobe Systems). Signals from different fluorescent probes were taken in sequential scan settings. Several cells for each labeling condition were analyzed and representative results were reported. Examinations were performed on either unfixed or fixed and permeabilized cells, as previously described [21, 24]. Flow-cytometry analyses were performed as reported [21, 40]. Several cells were analyzed for each labeling condition and representative examples are shown.

### Real-time polymerase chain reaction (qRT-PCR)

Total RNA was isolated from cultured cells using RNeasy^®^ Mini Kit (Qiagen) according to the manufacturer's instructions, quantified using Thermo Scientific NanoDrop^®^ ND-100, and then converted to cDNA using cDNA Synthesis Kit (Bioline, London, UK). HER2 expression was determined using 5′–AGTACCTGGGTCTGGACGTG–3′ (forward) and 5′–CTGGGAACTCAAGCAGGAAG -3′ (reverse) as primer sequences. PCR reactions were prepared using 2.0 μL of cDNA diluted in SensiMix™ SYBR kit (Bioline). qRT-PCR analysis was performed using the Applied Biosystems 7300-HT Real-Time PCR System. Levels of genes mRNA were expressed in relative copy numbers normalized against the housekeeping gene glyceraldehyde 3-phosphate dehydrogenase (comparative C_t_ method, 2^–ΔΔCt^).

### Animal model and *in vivo* magnetic resonance imaging (MRI) and spectroscopy (MRS) examinations

All animals were housed and treated in accordance with protocols approved by institutional authorities, in agreement with the European Community Directives (2013/63/EEC) and the Italian Law.

SKOV3.ip xenografts were subcutaneously implanted in 4–5 week old immunodeficient (SCID) female mice (Harlan Udine, Italy) as described [26]. MRI and MRS analyses were conducted at 4.7 T on a Varian/Agilent Inova horizontal bore system (Agilent, Palo Alto, USA) using a volume coil as transmitter and a surface coil as receiver (RAPID Biomedical, Rimpar, Germany) according to a reported protocol [26, 27]. Further details are reported in Supplementary Methods.

Histological analysis of xenograft sections following hematoxylin/eosin (H&E), Ki67 antigen labeling index (for tumor cell proliferation) and HER2 staining was performed on *ex vivo* specimens [27].

### High-resolution ^1^H MRS analyses of tissue extracts

The experiments were performed on aqueous tissue extracts either at 16.4 or 9.4 T using Bruker AVANCE spectrometers (Karlsruhe, Germany) as described [55, 56].

### Statistical analyses

Data were analyzed using GraphPad Software version 3.03. Statistical significance of differences was determined by Student's t test or by one-way ANOVA or repeated measurements ANOVA when specified. Differences were considered significant at *P* < 0.05.

## SUPPLEMENTARY MATERIALS FIGURES



## Abbreviations

Ab: antibody
ADC: apparent diffusion coefficient
Akt: protein kinase B or PKB
BrdU: 5-bromo-2′-deoxyuridine
CAV-1: caveolin-1
CLSM: confocal laser scanning microscopy
DAG: diacylglycerol
DAPI: 4′,6-diamidino-2-phenylindole
EGFR: epidermal growth factor receptor 1 (or HER1)
EOC: epithelial ovarian cancer
ErbB: human epidermal growth factor receptors family
FITC: fluorescein isothiocyanate
H&E: hematoxylin and eosin
HER2: human epidermal growth factor receptor 2
MAPK: mitogen activated protein kinase
MFI: mean fluorescence intensity
MR: magnetic resonance
MRI: magnetic resonance imaging
MRS: magnetic resonance spectroscopy
mAb: monoclonal antibody
mTOR: mammalian target of rapamycin
pAb: polyclonal antibody
pAkT: phosphorylated AkT
PC-PLC: phosphatidylcholine-specific phospholipase C
pHER2: phospho-HER2
PI: propidium iodide
PI3K: phosphoinositide 3-kinase
PTEN: phosphatase and tensin homolog
RECIST: Response Evaluation Criteria in Solid Tumors
STR: short tandem repeat (analysis)
tCho: total choline-containing metabolites
TRTZ: trastuzumab
VSF: vascular signal fraction
