# Supplementary material for: Phosphatidylcholine-specific phospholipase C inhibition reduces HER2-overexpression, cell proliferation and in vivo tumor growth in a highly tumorigenic ovarian cancer model
Source: Oncotarget. 2017 Jul 5;8(33):55022–38. doi: 10.18632/oncotarget.18992 (PMC5589638; doi:10.18632/oncotarget.18992)
Supplement: Supplementary file 1 [file oncotarget-08-55022-s001.pdf]

Phosphatidylcholine-specific phospholipase C inhibition reduces HER2-overexpression, cell proliferation and *in vivo* tumor growth in a highly tumorigenic ovarian cancer model

Supplementary Materials

SUPPLEMENTARY RESULTS

During the 9 days of D609 (or saline) treatments, all mice appeared to be healthy and there were no obvious signs or symptoms of drug toxicity during treatment and up to 12 days after the last scheduled drug administration. The animal weight was not significantly different between the groups at any time point . No obvious abnormalities were detected upon gross observation of the main organs in D609-treated animals.

In the pre-treatment tumors, as expected, no effects nor interactions of treatment were found. In the post-treatment volumes, ANOVA revealed an effect of treatment, $F(1,16) = 7.10$, $P = 0.02$, and an effect of time, $F(5,80) = 46.743$, $P < 0.0001$. Tukey HSD multiple posthoc comparisons evidenced a significant difference between the volumes of SAL and D609 treated tumors at all-time points except for 13 and 16 dpi.

We analyzed tumor volumes by dividing the treated tumors in the strong (D609-SR) and weak responder (D609-WR) subgroups [two-way repeated measures ANOVA, with treatment (SAL, D609-SR, D609-WR) x time (7 points) design]. As expected, no effects nor interactions of treatment were found in the pre-treatment tumors. In the post-treatment tumor volumes, ANOVA revealed an effect of treatment, $F(2,15) = 4.929$, $P = 0.02$, and an effect of time, $F(5,75) = 40.039$, $P < 0.0001$.

Tukey HSD multiple posthoc comparisons evidenced a significant difference between the volumes of SAL and D609-SR treated tumors at all-time points except for 13 dpi. No significant differences were instead observed for D609-WR and SAL or D609-SR.

SUPPLEMENTARY MATERIALS AND METHODS

Cells

SKOV3 and the in vivo passaged SKOV3.ip cells were cultured *in vitro* in RPMI supplemented with 10% (v/v) FCS. All cells were maintained in a humidified atmosphere at 5% CO_2 at 37 °C and routinely tested for absence of Mycoplasma infection using the Mycoplasma detection kit for conventional PCR Venorâ GeM (Minerva Biolabs GmmH, Berlin, Germany).

The *in vivo* passage used to obtain the SKOV3.ip cell line has been shown to represent a fundamental step for effectively developing EOC xenografts in immunodeficient mice using a tumor cell line [26].

Antibodies and reagents

Rabbit polyclonal antibodies (pAbs) raised against bacterial (*Bacillus cereus*) PC-PLC and selectively cross-reacting with mammalian PC-PLC [52] were obtained and characterized as reported [40, 53]. The anti-HER2 mAb W6/100 was a gift from Dr. PG Natali, Rome, Italy. Rabbit polyclonal anti-HER2 and anti-EGFR Abs were from Santa Cruz Biotechnology (Santa Cruz, CA, USA); mAbs against-phospho-HER2 (pHER2, Tyr1221/Tyr1222), phospho-MAPK (ERK 1/2, Thr202/Tyr204), caveolin 1, β integrin, Akt, phospho-Akt, and mTOR were from Cell Signaling Technology. The anti-β-actin, mAb was from Sigma-Aldrich and BD Biosciences, respectively. Trastuzumab (Herceptin®) and the protease-inhibitor cocktail was from Hoffman-La Roche. The FITC-conjugated anti-BrdU mAb was from BD Biosciences.

Alexa Fluor-488 and -594 conjugated $F(ab)_2$ fragments of goat anti-rabbit and anti-mouse IgG (H+L) Abs were purchased from Life Technologies and FITC-conjugated goat anti-human Abs from Cappel Co were used as secondary Abs. Goat anti-mouse and goat anti-rabbit IgG horseradish peroxidase (HRP)-conjugated Abs were supplied by BioRad Laboratories Inc).

Triton X-100, propidium iodide, D609, 5-bromo-2'-deoxyuridine; β-actin, and all other chemicals and biochemicals were from Sigma-Aldrich, unless otherwise specified.

Treatment of *in vitro* cultured cells

For immunofluorescence and flow cytometry analyses, cells were equally seeded at a density of 11.5×10^3 cells/cm^2, cultured for 24 h and then incubated

for 24, 48 and 72 h in complete medium in the presence or absence of either D609 (50 µg/mL), trastuzumab (10 µg/mL) or their combination.

For proliferation/viability assays, cells were cultured in 35 mm dishes and incubated at 37°C for 24 h and then exposed to D609, trastuzumab or their combination for 24, 48 or 72 h. Cell counting and cell viability (by trypan blue staining) were repeated at least three times with independent cell preparations.

Separation of lipid raft and non-raft fractions by sucrose gradient

Cells grown in T175 flasks were washed in cold PBS, lysed in 1% Triton X-100 containing buffer (25 mM MES, pH 6.5, and 150 mM NaCl), and the lysate was mixed 1:1 with 80% sucrose solution. Gradient fractionation (5–30% sucrose) and protein separation was carried out as previously described [21, 24, 40]. After ultracentrifugation at 100,000 g for 17 h in SW60 Sorvall rotor, twelve 375 µL fractions were collected from the top of the gradient and a sample was taken from each fraction for analysis by SDS-PAGE, in parallel with that of total cell lysate (30 µg total protein). The distribution of HER2 and PC-PLC in the gradient fractions was assessed by separation in 7% SDS-PAGE followed by Western blot.

Implantation, treatment of SKOV3.ip xenografts in immunodeficient mice

Cells (1×10^6) were subcutaneously injected into the dorsum of SCID mice and tumor volumes were monitored twice a week by caliper from early tumor detection until animal sacrifice. When tumors reached an average volume of about 160 mm^3 (which typically occurred 7 days post cell injection (dpi), mice (N = 23) were randomized into a treatment (N = 13) and a control group (N = 10) and intraperitoneally treated daily for 9 days with either D609 (1 mg/mouse/day) or saline (SAL).The volume specific growth rates were calculated according to ref. [33].

In vivo MR examinations

In vivo MR examinations were performed during treatment (from day 7 to day 15 after implantation) and tumor growth was further monitored up to 27 dpi. Tumor volumes were analyzed with a two-way repeated measures ANOVA, with treatment (SAL, D609) x time (7 points) design. To make tumor variances homogeneous at all time points, a square root transformation was applied to data. Time was a within subject factor, the treatment was a between subject factor. Pre- (1 time point) and post-treatment volumes (6 time points) were analyzed separately. Post hoc analyses were conducted using Tukey HSD tests.

Magnetic resonance imaging (MRI) and spectroscopy (MRS) analyses were conducted at 4.7 T on a Varian/Agilent Inova horizontal bore system (Agilent, Palo Alto, USA) using a volume coil as transmitter and a surface coil as receiver (RAPID Biomedical, Rimpar Germany) according to a protocol described in ref. 26. Briefly, animals were anaesthetized with isoflurane 1.5–2.0% in O_2 1 L/min. Anatomical (T1-weighted, T2-weighted) and diffusion-weighted MRI were acquired as described in ref. [26]. The apparent diffusion coefficient and vascular signal fraction (VSF) parameters were derived as described in ref. [51]. *In vivo* MRS protocol (PRESS, TR/TE = 4000/23 ms) which included T2-corrected water as internal reference and LCModel fitting routine was adopted for metabolite quantification. MR parameters, (tCho, ADC, VSF, T2 kurtosis, skewness) and specific growth rate were analysed with a one-way ANOVA. Post hoc analyses were conducted using Bonferroni test. Two mice per group were sacrificed during treatment and their tumors collected for histopathological and high resolution MRS analyses. The other mice were sacrificed between 10 and 15 days after the end of treatment. All dissected tissues were immediately frozen in liquid nitrogen.

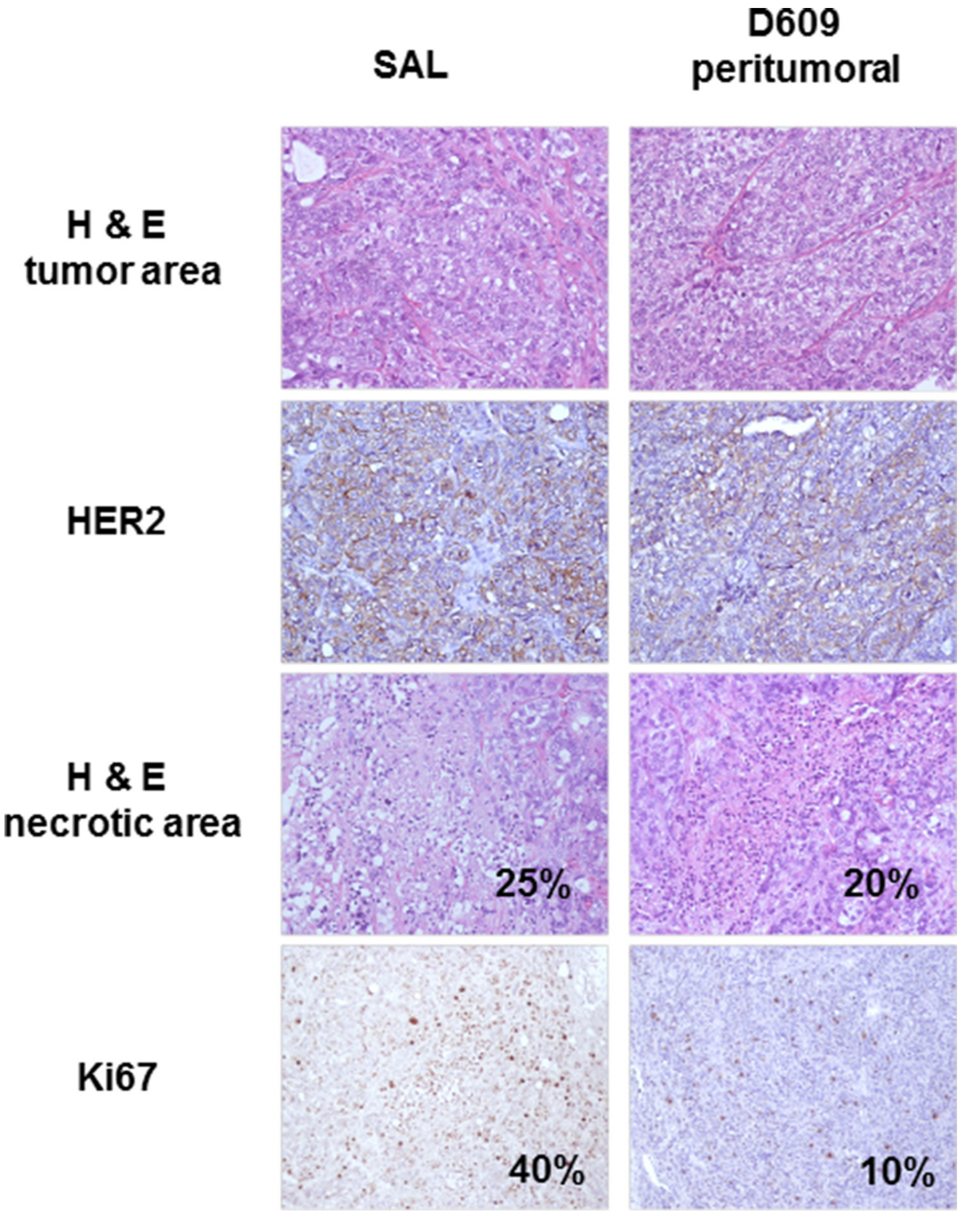

Supplementary Figure 1: Histological characterization by a) haematoxylin and eosin (H&E) staining; b) antigen proliferation index Ki67; and c) HER2 expression of *ex vivo* tumor xenograft samples explanted after 3 doses of weekly peritumoral D609 (1 mg/mouse) or saline (SAL-) treatments. HER2 staining was classified as strong/moderate in SAL-treated and weak in D609-treated tumor specimen sections.
